# The Limits of Two-Year Bioassay Exposure Regimens for Identifying Chemical Carcinogens

**DOI:** 10.1289/ehp.10716

**Published:** 2008-06-30

**Authors:** James Huff, Michael F. Jacobson, Devra Lee Davis

**Affiliations:** 1 National Institute of Environmental Health Sciences, National Institutes of Health, Department of Health and Human Services, Research Triangle Park, North Carolina, USA; 2 Center for Science in the Public Interest, Washington, DC, USA; 3 Center for Environmental Oncology, University of Pittsburgh Cancer Institute, Department of Epidemiology, Graduate School of Public Health, Pittsburgh, Pennsylvania, USA

**Keywords:** animal cancer tests, aspartame, bioassay designs, bisphenol A, cadmium, carcinogenicity, chemical carcinogens, genistein, toluene, toxicology

## Abstract

**Background:**

Chemical carcinogenesis bioassays in animals have long been recognized and accepted as valid predictors of potential cancer hazards to humans. Most rodent bioassays begin several weeks after birth and expose animals to chemicals or other substances, including workplace and environmental pollutants, for 2 years. New findings indicate the need to extend the timing and duration of exposures used in the rodent bioassay.

**Objectives:**

In this Commentary, we propose that the sensitivity of chemical carcinogenesis bio-assays would be enhanced by exposing rodents beginning *in utero* and continuing for 30 months (130 weeks) or until their natural deaths at up to about 3 years.

**Discussion:**

Studies of three chemicals of different structures and uses—aspartame, cadmium, and toluene—suggest that exposing experimental animals *in utero* and continuing exposure for 30 months or until their natural deaths increase the sensitivity of bioassays, avoid false-negative results, and strengthen the value and validity of results for regulatory agencies.

**Conclusions:**

Government agencies, drug companies, and the chemical industry should conduct and compare the results of 2-year bioassays of known carcinogens or chemicals for which there is equivocal evidence of carcinogenicity with longer-term studies, with and without *in utero* exposure. If studies longer than 2 years and/or with *in utero* exposure are found to better identify potential human carcinogens, then regulatory agencies should promptly revise their testing guidelines, which were established in the 1960s and early 1970s. Changing the timing and dosing of the animal bioassay would enhance protection of workers and consumers who are exposed to potentially dangerous workplace or home contaminants, pollutants, drugs, food additives, and other chemicals throughout their lives.

Additional research on molecular end points and other biological approaches has been recommended for evaluating the toxicity of chemicals (Committee on Toxicity Testing and Assessment of Environmental Agents 2007). However, currently and for the foreseeable future, short- and long-term bioassays represent the most reliable way to evaluate and profile the toxicology of chemicals to which humans are exposed, especially for predicting and preventing long-term adverse exposure effects, including cancer. We focus here on two potentially important means of increasing the sensitivity of carcinogenesis bioassays for identifying potentially carcinogenic food additives, pesticides, workplace chemicals and contaminants, pharmaceuticals, industrial chemicals, consumer products, and other natural and synthetic chemicals.

Chemical carcinogenesis bioassays in animals have long been recognized and accepted as valid predictors of potential cancer hazards to humans (Huff 1999, 2002; Rall 2000; Tomatis 2006; Tomatis and Huff 2002). The relevance of experimental bioassays to humans rests on four well-accepted observations: *a*) Experimental animals and humans are mammals sharing many basic genetic, pharmacologic, toxicologic, and carcinogenic responses; *b*) findings from independently conducted bioassays on the same chemicals are consistent; *c*) all known human carcinogens that have been tested adequately are also carcinogenic in animals and, almost without exception, share identical target sites; and *d*) nearly one-third of human carcinogens were first discovered to induce cancer in animals (e.g., 1,3-butadiene, diethylstilbestrol, dioxins, ethylene oxide, 2-naphthylamine, formaldehyde, vinyl chloride), although most of these were not regulated until human evidence mounted. Thus, in light of the fact that animal bioassays predict human cancer risks, conducting more sensitive tests would better protect the public, and especially workers, from involuntary exposure to animal carcinogens.

## Equivocal Carcinogenesis Bioassay Results

Worldwide, most carcinogenesis bioassays expose rodents for ≤ 2 years, starting after birth and ending well before natural death, which typically occurs for rats at 3 years and for mice at 2.5 years. In fact, as examples outlined below indicate, timing and duration of exposure and observation in chemical carcinogenesis bioassays may determine whether the results are positive or negative.

### Aspartame

Two 3-year-long lifetime feeding studies using Sprague-Dawley rats, conducted by the European Ramazzini Foundation of Oncology and Environmental Sciences in Bologna, Italy, have raised questions about the safety of the artificial sweetener aspartame (Abdo et al. 2007; Belpoggi et al. 2006; Huff and LaDou 2007; Soffritti et al. 2006, 2007). The first study began exposures at 8 weeks of age (postnatal study), whereas the second began exposures *in utero*. Exposures in both studies continued until the animals’ natural deaths, with the oldest rats living about 2.75–3.0 years. In the postnatal lifetime exposure study, aspartame caused mainly leukemias and lymphomas in males and females, peripheral nerve schwannomas in males, and possibly increases in total malignant tumors, kidney tumors, and brain tumors (Soffritti et al. 2006). The prenatal lifetime study showed increased incidences of lymphomas/leukemias in males and females, mammary gland cancers in females, and total tumor-bearing males (Soffritti et al. 2007). [The European Food Safety Authority and the U.S. Food and Drug Administration (FDA) dispute the conclusion of the first study and have not yet reviewed the second.] Previous 2-year feeding studies in Wistar rats did not detect increased tumor incidences (Ishii et al. 1981), although there was some debate about the occurrence of rare tumors of the brain, also observed in the Ramazzini studies, and there are general concerns regarding the questionable quality of other earlier negative studies (Davis 2007).

### Cadmium

Cadmium is another telling example of a chemical that was not shown to be carcinogenic in 2-year studies of Wistar rats (Löser 1980), but caused various tumors in the lung in a 31-month study of Wistar rats (Takenaka et al. 1983). According to Takenaka et al. (1983), “length of experimental duration was the likely reason” their study, but not the other study, showed positive carcinogenicity results. Interestingly, Löser (1980) exposed animals to cadmium for 24 months, about 6 months longer than did Takenaka et al. (1983), who exposed animals for 18 months but observed them 13 months longer than did Löser.

### Toluene

**Figure 1:**
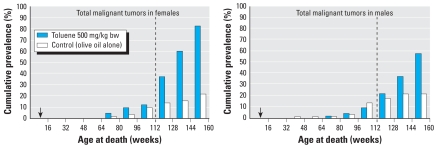
Cumulative prevalence of total malignant tumors in females and males histopathologically observed, by age at death, among Sprague-Dawley rats treated with 500 mg/kg body weight/day of toluene for 104 weeks and then kept under control conditions until death or 130 weeks (experiment BT 903). Dashed lines indicate 112 weeks, the typical termination of NTP studies. Arrows indicate beginning of exposure. Reproduced from Soffritti et al. (2004) with permission from the European Ramazzini Foundation.

Soffritti et al. (2004) orally treated Sprague-Dawley rats with toluene for 2 years and observed them for an additional 6 months. At the end of this observation period, significant increases occurred in several tumors, including total malignant tumors (Figure 1); tumors of subcutaneous tissue, mammary glands, and oral cavity, lips, and tongue; and lymphomas/leukemias. In contrast, the National Toxicology Program’s (NTP) 2-year inhalation bioassays in Fischer rats and B6C3F_1_ mice did not detect any chemically related tumors (Huff 2003). Thus, the 26 extra weeks of observation without additional exposure in the Sprague-Dawley study (Soffritti et al. 2004) allowed time for tumors to develop, progress, and become detectable. An animal strain–response difference between laboratories is possible, but this was not the case in all other comparisons of carcinogenicity studies performed by the Ramazzini Foundation and the NTP, nor have such differences been shown generically (Huff 2002).

## Discussion

### Advantages of beginning exposure during intrauterine development

Over the years, the NTP has tested chemicals for possible carcinogenicity in about 600 2-year bioassays, most often using both sexes of two strains of rodents, rats and mice. Yet only 7 (~ 1%) of those involved *in utero*/perinatal exposures:

- Azidothymidine transplacental carcinogenesis study (NTP 2006)
- *t*-Butylhydroquinone (TBHQ) (NTP 1997a)
- D&C yellow no. 11 (NTP 1997b)
- 5,5-Diphenylhydantoin (phenytoin) (NTP 1993a)
- Ethylene thiourea (NTP 1992)
- Genistein (NTP 2007)
- Polybrominated biphenyl mixture (Firemaster FF-1) (NTP 1993b).

The developing fetus is particularly sensitive to effects of hormones and estrogenic (and other) chemicals. For instance, prenatal exposure of humans to diethylstilbestrol (DES) provides a classic example of transplacental carcinogenesis predicted in animals (Newbold and McLachlan 1982) and confirmed in humans (Herbst et al. 1971; Palmlund 1996; Palmer et al. 2006). Because exposures to phytoestrogens and synthetic hormonally active compounds, such as bisphenol A (BPA), are increasing in diets of infants and children, these materials need to be investigated in prenatal and perinatal bioassays. Newbold et al. (2001) evaluated the endocrine disruptor genistein in a 5-day neonatal exposure in CD-1 mice and after 18 months found increases in adenocarcinomas of the uterus. They then designed expanded bioassay with three exposure regimens: *a*) continuous exposure from conception through 2 years of age; *b*) exposure from conception through 20 weeks (140 days) followed by a control diet to 2 years of age; and *c*) dosing from conception through weaning (21 days) followed by the control diet to 2 years. Increases in tumors (mammary gland and pituitary gland tumors) were observed only in the first experiment.

An *in utero* exposure protocol used to study effects of TBHQ (Abdo and Kari 1996; NTP 1997a) 2.5 years after exposure began should be considered by the NTP and regulatory agencies as a model for cancer bioassays. Female F_0_ rats were fed diets containing vary-ing amounts of TBHQ, beginning 2 weeks before cohabitation with males and continuing through gestation and lactation until F_1_ pups were weaned. F_1_ rats continued to receive postweaning diets containing TBHQ for 123 weeks for males and 129 weeks for females. Interestingly, despite this inclusive exposure protocol from conception up to nearly 30 months of age, there was no evidence of carcinogenicity. In fact, decreases in mammary gland tumors occurred in both sexes. These findings certainly contradict the notion that studies using *in utero* exposure with follow-up for about 2.5 years invariably result in carcinogenic activity.

In contrast, recent studies of the widely used plasticizer additive BPA indicate that pre-natal exposures can induce obesity in offspring (Dolinoy et al. 2007), a phenomenon that has not been observed with postnatal exposures alone. Prenatal exposure of pregnant Agouti Avy mice to BPA, which is a major component of polycarbonate plastics and epoxy resins, significantly reduced DNA methylation (Dolinoy et al. 2007). Others have reported that prenatal BPA also increases mammary tumor development and other developmental abnormalities (Soto et al. 2008), and may increase recurrent miscarriages (Sugiura-Ogasawara et al. 2005). Thus, experiments of prenatal exposure to BPA find that such exposures imprint mammary cells, leaving them especially sensitized to later cell growth and hormonal stimulation—characteristics also found in tumors. It may also be relevant that low levels of BPA have been found to activate genes in noncancerous breast cells in a way that mimics that seen in highly aggressive breast cancer (Dairkee et al. 2008), indicating that BPA plays a critical role both prenatally and postnatally.

### Advantages of studies with longer observation periods versus 2-year studies

Experimental studies should be designed with optimum sensitivity to identify likely adverse health problems throughout humans’ increasing life span. Humans, of course, consume or are exposed to countless natural and synthetic substances during gestation, nursing, and the rest of their lives. In modern societies, proportionally more people are living until their 70s, 80s, and 90s, long after prenatal and childhood exposures and retirement from workplace exposures. Because most long-term rodent carcinogenesis studies do not involve *in utero* exposure and are intentionally terminated after 2 years (104 weeks) of exposure, they cannot shed light on the effects of chemicals on embryos/fetuses/neonates or “elderly” animals. Likewise, studies truncated after 2 years of exposure do not allow sufficient latency periods for late-developing tumors, such as the 80% of all human cancers that occur after 60 years of age. Because a 2-year-old rat is roughly equivalent to a 60- to 65-year-old person, conventional 2-year-long bioassays cannot detect tumors that will develop later in life.

Although some researchers have suggested reducing the duration of rodent studies to 18 months (e.g., Davies et al. 2000), scientists at the National Institute of Environmental Health Sciences reject that recommendation, noting that animals in such studies would be the equivalent of humans only 30–50 years of age and would reduce statistical power (Bucher 2002; Haseman et al. 2001; Kodell et al. 2000). Instead, a number of researchers have recommended extending the duration of rodent studies to ≥ 30 months (Davis 2007; Haseman et al. 2001; Huff 2002; Huff et al. 2007; Maltoni 1995; Soffritti et al. 2002). In some cases, as the Ramazzini Foundation sometimes does, exposure to industrial chemicals is stopped after 2 years (“retirement age”), and the animals are then observed until ≥ 30 months of age to determine delayed or persistent carcinogenic activity (Maltoni 1995; Soffritti et al. 2002). Ceasing exposure at 2 years without monitoring tumor development for additional time cannot estimate the impact of food additives, drugs, and other chemicals on humans who die in their 70s or later.

### Advantages of 2-year studies over studies with longer observation periods

Two-year studies do have certain advantages over longer studies. First, because these studies are standard protocols and designs, a great deal of experience has been gained from them, including information on tumor formation in historical controls.

Second, these briefer studies yield somewhat quicker results, which is useful for protecting public health.

Third, shorter tests are somewhat, but not proportionately, cheaper to conduct. However, because of the numerous fixed costs of animal studies (animals, postmortem pathology, etc.), longer studies are not commensurately marginally costlier. In any case, economy should not trump sensitivity and the reliability of results.

Ancillary costs of longer studies include educating researchers on how to conduct them and carrying out more frequent inspections at the later stages for moribund or sickly animals to minimize autolysis in animals that die naturally at night and may not be discovered immediately. However, certain tissues are affected more than others, and tumors would remain relatively inviolate; moreover, even in 2-year studies, only 60–70% of animals remain alive at the end.

Fourth, some tumor rates increase, and old-age lesions may complicate discerning nonneoplastic effects.

Finally, whereas tumor incidences in various strains of control animals are known on the basis of numerous studies, it would take time to develop a body of data on tumor incidence and other abnormalities for these longer time periods.

Some researchers have argued that higher rates of spontaneous tumors and other factors would undermine the value of longer-term studies (Solleveld and McConnell 1985), but those assertions are not based on actual bio-assays—comparing, for instance, the relative abilities of 24-month, 30-month, and lifetime bioassays to detect the effects of known carcinogens. Most important, any “savings” accruing from current testing protocols are only illusory if false-negative results lead to the exposure of millions of people to substances that caused cancer or other maladies.

To maximize the knowledge gained from costly full-lifetime studies, protocols should be expanded to provide for periodic sacrificing to determine time-to-tumor and biological sampling to determine internal doses, metabolite levels, genetic alterations, and other data relevant to characterizing the pharmacokinetic and pharmacodynamic activity of toxicity and noncancer disease.

## Conclusion

The evidence presented here indicates that extending animal bioassays beyond 2 years and beginning exposure *in utero*, especially for endocrine-disrupting chemicals that “act” preferentially in early life (Huff 1996, 2001; Huff et al. 1996; Newbold et al. 2001; Newbold and McLachlan 1982; Thayer and Foster 2007; Tomatis et al. 1992; Yamasaki et al. 1992), would provide more reliable and appropriate indicators of human risk. The FDA, U.S. Environmental Protection Agency, Occupational Safety and Health Administration, Consumer Product Safety Commission, NTP, and others should reconsider their designs of “best practices” regarding long-term studies of chemicals for detection of carcinogenic risks to humans. We urge those governmental agencies, perhaps in conjunction with such other agencies as the World Health Organization’s International Agency for Research on Cancer, to conduct and compare the results of 2-year and longer-term feeding studies, with and without *in utero* exposure, of known or equivocal-evidence carcinogens.

It is noteworthy that the FDA already “recommends that *in utero* exposure be included in carcinogenicity studies due to the fact that exposure to food ingredients occurs during all stages of life” (FDA 2006). If bio-assays demonstrate that studies longer than 2 years and/or with *in utero* exposure are better able to identify carcinogens, regulatory agencies should revise their testing guidelines, most of which were established > 40 years ago (Bucher 2002; Huff and Moore 1984; Weisburger 1983).

Another gap in regulatory protocols involves male-mediated teratogenesis—the study of compounds’ capacity to impede reproductive health of offspring through paternal genomic and other male-mediated routes (Davis et al. 1992). Regarding endocrine disruptors and other chemicals that cross the placenta and affect critical windows of development, one need not wait for comparison bio-assays before routinely conducting *in utero* and longer-term bioassays.

Benefits to public health from conducting more sensitive bioassays that reflect early life exposures and continue throughout a natural lifetime of observation could be considerable.
